# Tele-Follow-Up of Older Adult Patients from the Geriatric Emergency Department Innovation (GEDI) Program

**DOI:** 10.3390/geriatrics4010018

**Published:** 2019-01-29

**Authors:** Lucy Morse, Linda Xiong, Vanessa Ramirez-Zohfeld, Scott Dresden, Lee A. Lindquist

**Affiliations:** 1 Division of General Internal Medicine & Geriatrics, Northwestern University Feinberg School of Medicine, Chicago, IL 60611, USA; Lucy.Morse@northwestern.edu (L.M.); Linda.Xiong@northwesten.edu (L.X.); Vanessa-Ramirez-0@northwesten.edu (V.R.-Z.); 2 Department of Emergency Medicine, Northwestern University Feinberg School of Medicine, Chicago, IL 60611, USA; S-Dresden@northwestern.edu

**Keywords:** Emergency department, follow-up phone calls, older adults

## Abstract

The objective of this study was to characterize the content and interventions performed during follow-up phone calls made to patients discharged from the Geriatrics Emergency Department Innovation (GEDI) Program and to demonstrate the benefit of these calls in the care of older adults discharged from the emergency department (ED). This study utilizes retrospective chart review with qualitative analysis. It was set in a large, urban, academic hospital emergency department utilizing the Geriatric Emergency Department Innovations (GEDI) Program. The subjects were adults aged 65 and over who visited the emergency department for acute care. Follow-up telephone calls were made by geriatric nurse liaisons (GNLs) at 24–72 h and 10–14 days post-discharge from the ED. The GNLs documented the content of the phone calls, and these notes were analyzed through a constant comparative method to identify emergent themes. The results showed that the most commonly arising themes in the patients’ questions and nurses’ responses across time-points included symptom management, medications, and care coordination (physician appointments, social services, therapy, and medical equipment). Early follow-up presented the opportunity for nurses to address needs in symptom management and care coordination that directly related to the ED admission; later follow-up presented a unique opportunity to resolve sub-acute issues that were not addressed by the initial discharge plan and to manage newly arising symptoms and patient needs. Thus, telephone follow-up after emergency department discharge presents an opportunity to better connect older adults with appropriate outpatient care and to address needs arising shortly after discharge that may not have otherwise been detected. By following up at two discrete time-points, this intervention identifies and addresses distinct patient needs.

## 1. Introduction

Providing affordable, quality health care to older adults in the emergency department (ED) is of paramount concern in the present and future medical landscapes. The number of adults ages 65 and over in the United States (US) is projected to more than double in the next 40 years, comprising nearly 25% of the total population by 2069 [1]. This older adult population is more likely to be admitted to the hospital following a visit to the emergency department relative to younger adults, and nearly half are readmitted within six months. Moreover, hospital admission is both costly and associated with a number of adverse outcomes for older patients, especially [2,3,4]. Several novel interventions have been designed to identify at-risk older adults interfacing with the emergency department in an effort to streamline impactful care and to reduce the financial and personnel burdens that accompany the hospital admission of older adults [5,6,7].

One such point of intervention is avoidable admission for reasons other than acute illness, including functional decline, polypharmacy, dementia, and an unstable living environment. Although these conditions may not be grounds for admission independently, in combination with acute illness and complex medical needs, they may lead to imminent risk to the patient if discharged [8,9].

### 1.1. The Geriatric Emergency Department Innovation (GEDI) Program

The GEDI program was developed to optimize the treatment of older adult patients entering the emergency department in order to shorten ED visits and reduce hospital admissions, while connecting patients with the social and outpatient medical services best suited to their needs [9]. At Northwestern Memorial Hospital (NMH), nurses specially trained in geriatrics—geriatric nurse liaisons (GNLs)—identify older adults through the Identify Seniors at Risk (ISAR) tool and perform further screens for common geriatric syndromes by assessing for cognition (Short Portable Mental Status Questionnaire), delirium (Confusion Assessment Method), functional status, (Katz Activities of Daily Living), fall risk (Timed Up and Go test), caregiver strain (Modified Caregiver Strain Index), and transition readiness (Care Transitions Measure-3) [9,10]. GNLs assess the patient and determine if the patient can receive optimal treatment at home with outpatient follow-up instead of being admitted to the hospital. Hospitalization is well-established to be detrimental for older adults with resulting decreased physical function, worsening cognition, medication issues, and a multitude of other adverse effects. Based on results from the GNL testing series, patients are referred to primary care and specialist services, physical therapy, occupational therapy, and social and home care services as appropriate. The GNL designs a care plan for discharge, and then, the patient is sent home from the ED. After discharge, the GNL makes follow-up phone calls at two time-points following the ED admission to assess the continuing needs in care and answer any medical or logistical questions that may have arisen since the ED discharge [9,10].

The results from NMH’s GEDI program have shown that the intervention results in a significant decrease in the hospital admission of older adults presenting to the emergency department [9]. Individual components of the GEDI program’s effectiveness and impact, however, remain to be analyzed. Specifically, the impact of the telephone follow-up care provided to patients discharged from the GEDI program and ED has yet to be examined.

### 1.2. Follow-Up in Emergency and Ambulatory Care Settings

Previous research on the impact of follow-up care in the medical setting strongly indicates its important role in high-value, low-cost care. Follow-up phone calls made by pharmacists 48 h after hospital discharge at an academic medical center resulted in greater patient satisfaction, medication resolution, and medical referral for a subset of patients and fewer ED readmissions 30 days post-discharge for patients receiving the phone calls [11]. Similar interventions in pediatric, geriatric, and adult populations have demonstrated that calls can present an opportunity to assist a significant proportion of patients with medical questions and issues arising post-discharge and can increase patient adherence to appointments and care instructions in post-discharge and traditional clinic settings [12,13,14,15]. While the value of follow-up phone calls in the setting of the GEDI program at NMH is strongly indicated, the outcomes of these calls remain unexamined. Furthermore, the value of these follow-up calls in addressing patient needs as it relates to timepoint post-discharge remains, to our knowledge, unstudied in the context of care for older adults.

The objective of this study was to characterize the content and interventions performed during follow-up phone calls made to discharged patients who presented at the GEDI program academic medical center and to demonstrate the benefit of these calls to the care of older adults discharged from the emergency department.

## 2. Materials and Methods

### 2.1. Participants

All the patients over the age of 65 presenting to the emergency room and meeting the ISAR criteria for further work-up through the GEDI program received the follow-up phone calls that were the subject of this analysis. The data were anonymized prior to qualitative analysis. This project was considered exempt by the Northwestern University Institutional Review Board.

### 2.2. Data Collection

GEDI follow-up phone calls were made at two time-points: 24–72 h and 10–14 days following the primary ED admission. The GNL responsible for the patient case performed the follow-up phone call and transcribed the conversation in an open-response format; notes from the patient charts were abstracted, and these notes were the subject of the data analysis, until saturation of themes was achieved.

### 2.3. Data Analysis

The transcripts were then de-identified and analyzed by 3 authors (LL, LM, and LX) using content and constant comparative techniques [16], through which the coders independently assessed the participant responses for focal themes. The authors then convened to compare and compile their findings to create a list of major themes, which were discussed through further meetings, such that a consensus was reached in all the cases of initial discrepancy. The coders independently organized the content into an overarching categorical system: Multiple coders were allowed to independently develop categorical systems in order to control for the subjective bias each coder brings to the analytic process [17]. From these overarching categories, the coders synthesized the relevant themes and compared them across time-points.

## 3. Results

### 3.1. Obtaining Thematic Saturation

The responses to telephone follow-up were consecutively abstracted and qualitatively analyzed, until thematic saturation was reached (e.g., consensus of the research team that no new information was emerging in the data collection) [18,19,20]. Hematic saturation was determined to be completed after 57 charts. Prior qualitative research has shown that as few as 8–12 interviews are needed to reach thematic saturation [21,22].

### 3.2. Subject Characteristics

For the 57 subjects, the mean age was 88.6 years (range 66–96 years). The subjects had a mean ISAR score of 4 (range 1–6). From a cognitive perspective, the subjects scored a mean 1.65 (range 0–7) on the Short Portable Mental Status Questionnaire (SPMSQ, normal is less than 2). From a functional perspective, of the six basic activities of daily living (ADLs: eating, bathing, getting dressed, toileting, transferring, and continence), the subjects were able to do a mean of 5.28 (range 0–6) independently without assistance. These data were self-reported. A total of 14 subjects used an assistive device (cane or walker) and scored a mean of 15.5 s (range 8–34 s) on the Timed Up and Go test.

### 3.3. Key Themes

Key themes in the patients’ concerns and the nurses’ responses emerged in both time-points. The emergent themes in the patient concerns included clinical symptoms, medication questions, medical equipment, therapy or home health services, and follow-up with specialists or primary care providers (Table 1). The emergent themes in the nurse responses included providing clinical information, medication counseling, care coordination relating to appointments, and communication with social workers to arrange other social services (Table 2). These themes were largely overlapping across time-points; however, certain themes were unique to the later time point, and notably, certain themes were much more frequently encountered in one time point or the other. Upon further investigation of frequency disparities in theme across the time-points, certain content specificities within the overarching categories were found to vary, leading to the demonstrated differences.

### 3.4. Comparison across Timepoints

#### 3.4.1. Clinical Symptoms

Clinical symptoms made up a large portion of issues discussed at both the 24–72-h and 10–14-day time-points; however, the concerns differed in content within this theme. Clinical concerns raised shortly after discharge were typically found to relate to the concern for which the patient presented to the ED initially, as exemplified by this follow-up note: “*The patient discussed how long her eye might remain red...informed to follow-up with ophthalmologist or ED if increased discharge, draining, bleeding from the eye, pain, or visual disturbances*.” By contrast, symptom-related concerns at 10–14 days were sometimes unrelated to the cause for ED admission, as shown by this note: “*Has an appt. with primary care physician in another week. I asked her to speak with the doctor about her elevated blood pressure. Patient was concerned that it has not been controlled with her current medication but will seek guidance from primary care physician.*” A subset of clinical concerns presenting at the 10–14-day time-point was entirely unrelated to the chief concern at the ED admission: “*Complaining of productive cough and occasional wheezing. Using inhaler with some relief*”. By contrast, none of the clinical concerns identified at 24–72 h were entirely new or unrelated to the concern precipitating the ED visit. In short, while clinical/symptom-related concerns were a common feature of the follow-up calls at both time-points, the later time-point demonstrated use in identifying sub-acute or unrelated clinical concerns that had not been apparent at the earlier time-point.

#### 3.4.2. Physician Follow-Up Appointment Scheduling

Although follow-up physician scheduling was managed at both time-points, concerns and new information were more frequently elicited at the later time-point; while instructions were given to follow up and appointments were scheduled by the GNLs at the earlier time-point, the later time-point allowed the nurses to follow up on planned/recommended appointments that had not successfully been made, as shown in this note: “*Spoke with patient to verify that she called the ophthalmology clinic for an appointment...notified her that I would call back tomorrow to be sure she was ok and had made her follow-up appt...addendum: scheduled follow-up ophthalmology appointment for 4/23/13 at 1500*.”

#### 3.4.3. Durable Medical Equipment (DME), Physical Therapy (PT), and Home Health Nursing (HN) Coordination

Nurse coordination of durable medical equipment (DME), physical therapy (PT), and home health nursing (HN) took place more frequently at the 24–72-h follow-up relative to the 10–14-day follow-up. Early communication is critical for quick access to DMEs and HN services, such as home safety evaluations: “*Having difficulty obtaining walker, which was told was ordered 2 weeks ago, but home health agency said they are waiting on physician order. Will contact our social worker to help facilitate. 1445: Prescription obtained for walker from emergency department physician, faxed by the Geriatric Emergency Department Innovation (GEDI) nurse liaison and will facilitate walker delivery to patient.”*

However, calls at the second time-point added value in that they allowed nurses to connect patients with services that were identified as potentially beneficial upon the initial physician follow-up, as shown here: “*The patient states her primary care physician suggested physical therapy. Will call office to see about order for physical therapy*.”

## 4. Discussion and Conclusions

This study set out to characterize the content and interventions performed during follow-up phone calls made to discharged patients who presented at the GEDI program academic medical center and to demonstrate the potential benefit of these calls to the care of older adults discharged from the emergency department. Qualitative analysis revealed key emergent themes in both the patient concerns expressed during the calls, as well as the nurse responses to the patient needs raised during in the follow-up. Concerns regarding clinical symptoms, medications, specialist and primary care physician follow-up, and durable medical equipment, physical therapy, and home health nursing services arose frequently across both time-points. The nurses responded to such concerns by providing clinical symptom and medication counseling, confirming or scheduling physician follow-up appointments and interfacing with case managers in order to navigate patient access to durable medical equipment, physical therapy, and home health nursing services; these actions were taken by nurses at both time-points. Some patient concerns and nurse responses were only encountered at the later time-point, including caregiver coordination and management of transitions of care.

### 4.1. Significance of Cross-Timepoint Comparisons

While there was a great amount of consistency across time-points where the emergent themes were concerned, a variation of content within themes, as well as differences in frequency of theme presence across time points, was identified. Clinical concerns and symptom management at the 24–48-h time-point was consistently related to the chief concern that precipitated the ED intake, while this theme at the 10–14-day follow-up included newly identified clinical concerns, as well as issues that were sub-acute or secondary at the time of the ED admission. This finding suggests the added value of the later follow-up, as it has the potential to support the increased identification of underlying or newly arising patient needs that may have gone unmet without a later follow-up. Later follow-up also presented a unique opportunity for improved transitions of care and care management relating to physician follow-up. The later time-point appears to facilitate scheduling of appointments that were recommended but had not yet been initiated, which was a pattern more commonly observed at this stage. The early follow-up proved critical for establishing initial connections to medical equipment, therapy, and home nursing services; however, later follow-up also added value where apparent ‘missed connections’ with services were concerned.

In examining the patient concerns and interventions directly from the telephone calls, our qualitative results show that there is a benefit to telephone follow-up after an emergency department visit. While Biese et al. found no benefit of a telephone call to older adults from the ED on readmission or death in primary outcomes, our results show that there are unmet needs after a hospital visit that benefit from phone follow-up and interventions [23]. The singular outcomes of readmission or death may not provide a complete picture as to the effects or benefits of a telephone follow-up after an ED visit. Hwang et al. encouraged expanding the scope of the outcomes assessed, beyond hospital admissions and ED revisits, as a necessary starting point for future work—including how ED interventions affect important outcomes such as perceived social support, use of home care services and physician referrals, and functional decline [24]. Our results show that during telephone follow-up after an ED visit, issues relating to older adult patients that can be eased by essential interventions, such as home services, medical equipment, social support, and physician referrals, can be initiated to improve quality of care.

### 4.2. Strengths and Limitations

The qualitative design of this study allowed for the identification of novel patterns and themes that elucidate the value of two distinct time-points for follow-ups with patients admitted to the emergency department. The comparison of themes between two time-points illustrated the distinct benefits of each stage of follow-up. The study is limited, however, in that the nature of these data and methods do not permit for parametric statistical analysis; therefore, the frequencies with which these themes arose in early and late follow-up, while informative descriptively, cannot be applied to establish the significance of the observed differences.

### 4.3. Recommendations and Future Directions

Follow-up phone calls following an emergency department visit, occurring at two time-points, identified the questions and unmet needs of older adult patients after emergency department discharge. The geriatrics nurse liaisons (GNLs) were able to remedy these concerns and needs, thus establishing the value of follow-up phone calls within the context of the GEDI program. These findings suggest that both short-term and longer-term follow-up telephone calls are critical, as they each promote the management of distinct problems by allowing the GNLs to identify needs that arise at different stages post-discharge.

A future focus for the investigation of GEDI follow-up will be establishing the impact of these telephone calls on quantitative, outcomes-based metrics, such as 30-day readmission. The readmission of older adults following emergency room discharge is a pressing issue for patients and hospitals, with rates of 30-day readmission estimated to reach between 12 and 20% [18,19]. Based on our findings, GEDI follow-up calls provide an opportunity to connect older adults with the care that they need and demonstrate concrete incidences of direct intervention taken by GNLs to promote patient access to medical care, as well as non-medical services. Important next steps in this line of research include demonstrating the downstream impacts of reaching patients in this way on outcome measures and using the newly identified themes to direct the improvement of discharge interventions, such as the GEDI program.

## Figures and Tables

**Table 1:** Patient concerns at follow-up: Patient concerns arising 24–72 h and 10–14 days post-discharge.

Emergent Themes	Selected Quotes
Clinical/symptom management	“The patient discussed how long her eye might remain red...informed to follow-up with ophthalmologist or emergency department if increased discharge, draining, bleeding from the eye, pain, or visual disturbances.”
Medication	“Taking acetaminophen only 2 times a day. Afraid of aggravating liver.” “Did quiz him about the use of his insulin and he states that his blood sugars have been in the 130-150 range. States he is checking his sugars two times a day”
Therapy, home health, or medical equipment	“Patient concerned about being alone and having to take shower without having someone check on her” “Questions in regard to when home health care and physical therapy would come to evaluate the patient”
Physician follow-up	“Has appointment on the 23rd. Will be receiving home occupational therapy in addition to home physical therapy. Patient lost list of geriatric psychiatrists, asked to resend to home. Mailed today.”
Transitions of care **	“Discharged home from skilled nursing facility on 4/18. Scheduled for blood draw and evaluation in the Coumadin Clinic today. Appointment with cardiologist scheduled for 4/29.”

** Theme only present at the 10–14-day follow-up. Quotes edited for grammar and clarity.

**Table 2:** Nurse response at follow-up: nurses’ responses to patient concerns identified at 24–72 h and 10–14 days post-discharge.

Emergent Themes	Selected Quotes
Symptom counseling	“Informed to elevate, apply ice or take Tylenol, as needed” “instructed on warm compresses and stretching”
Medication counseling	“Not discharged from emergency department observation unit with any pain medications. Encouraged acetaminophen 650 mg every 4 h as needed”
Physician care coordination	“Encouraged to follow-up with primary care physician (PCP) and Gastroenterologist as scheduled for increased pain” “... Physician referral services contacted and made appointment for patient for follow-up with primary care physician for Wednesday this week.”
Therapy, home health, or medical equipment coordination	“Having difficulty obtaining walker, which was told was ordered two weeks ago, but home care agency said they are waiting on physician order. Will contact our social worker to help facilitate. 1445: Prescription obtained for walker from emergency department physician faxed by the Geriatric Emergency Department Innovation (GEDI) nurse liaison and will facilitate walker delivery to pt.” “Patient required multiple calls to help facilitate outpatient physical therapy. Prescription faxed to rehabilitation center with diagnosis.”
Social work/services coordination	“...To have endoscopy and colonoscopy at the end of May. Looking into Medical Alert Systems. Given several names from Social worker list.”
Caregiver coordination **	“Patient states he is not aware of visits; caregiver daughter is in charge. Called daughter and left a message if she needs any assistance with appointment scheduling.”

** Theme only present at 10–14-day follow-up. Quotes edited for grammar and clarity.

## Impact Statement

We certify that this work is novel or confirmatory of recent novel clinical research. The potential impact of this research on clinical care or health policy includes the following: (1) Providing data supporting the use of follow-up phone calls in emergency department protocols for the care of older adults. (2) Identifying key care needs of older adults discharged from the emergency department that may be addressed through follow-up phone calls.
